# Exposure to models’ negative facial expressions whilst eating a vegetable decreases women’s liking of the modelled vegetable, but not their desire to eat

**DOI:** 10.3389/fpsyg.2023.1252369

**Published:** 2024-01-11

**Authors:** Katie L. Edwards, Jason M. Thomas, Suzanne Higgs, Jacqueline Blissett

**Affiliations:** ^1^School of Psychology, Aston University, Birmingham, United Kingdom; ^2^School of Psychology, University of Birmingham, Birmingham, United Kingdom

**Keywords:** young adults, facial expression, modeling, vegetable liking, desire to eat

## Abstract

**Introduction:**

Food enjoyment can be conveyed through facial expressions. Observing others’ enjoyment of food has been found to influence adults’ desirability of liked and disliked food. Exposing adults to other eaters enjoying nutritious foods that are typically disliked (e.g., vegetables) could enhance the consumption of vegetables by young adults. However, this remains to be examined in young adult populations. This study examined the effect of models’ facial expressions towards raw broccoli on young adult women’s change in liking and change in desire to eat a modelled vegetable (raw broccoli) and a non-modelled vegetable (cucumber).

**Methods:**

Young adult women (*N* = 205) were randomised to watch a video of unfamiliar adult models eating raw broccoli with a positive, negative, or neutral facial expression. Participants’ change in liking and change in desire to eat the modelled and non-modelled vegetable was examined.

**Results:**

Observing models conveying negative facial expressions whilst eating raw broccoli resulted in a statistically significant reduction in liking ratings of broccoli, but not cucumber. There was no effect of models’ facial expressions on the change in desire to eat foods.

**Discussion:**

These findings suggest that watching others express a negative facial expression whilst eating a raw vegetable reduces women’s liking of the modelled vegetable, in the absence of a significant change to their desire to consume these foods. This highlights the power of others’ negative facial expressions on food liking. Further work is needed to establish the effect of others’ facial expressions on vegetable intake.

## 1 Introduction

Most young adults consume fewer vegetables than recommended (Larson et al., 2012; Health Survey for England, 2018; Rodrigues et al., 2019). This is concerning given the associated health benefits of sufficient vegetable consumption (Boeing et al., 2012; Slavin and Lloyd, 2012). Young adulthood is associated with poor dietary behavior, such as lower fruit and vegetable intake (Nelson et al., 2008; Winpenny et al., 2018), thus research is warranted to understand how social influences affect young adults eating behavior. Dietary behaviors can persist into later adulthood (Craigie et al., 2011), therefore gaining an understanding about the influence of others’ food enjoyment on young adults’ eating behavior is important to identify novel ways to improve young adults’ eating behavior.

Modeling is a powerful and robust social influence on eating behavior (Cruwys et al., 2015; Vartanian et al., 2015). One factor that can influence the modeling of eating is observing a co-eater’s enjoyment of food, such as a verbal statement about how palatable a food is (e.g., “mmm, this is yummy”) or a facial reaction whilst eating the food (e.g., wrinkling your nose to a disliked taste). Social Learning Theory suggests that behaviors are more likely to be imitated if positive consequences are observed, and less likely to be imitated if negative consequences are observed (Bandura, 1977). For example, an observer may be less inclined to want a food after watching someone else look disgusted whilst eating it. Research has examined the effect of others’ facial expressions (FEs) toward food on adults’ perceived desirability of food. Barthomeuf and colleagues exposed adults to images of eaters looking at food with a pleasure, disgust, or neutral facial expression (FE). Findings showed that exposure to eaters looking at food with disgust FEs decreased the desire to eat liked food, whereas exposure to eaters looking at food with pleasure FEs increased the desire to eat disliked food (Barthomeuf et al., 2009, 2012). In addition, Soussignan et al. (2015) exposed adults to videos of avatars conveying joy, disgust or neutral FEs toward food. It was found that exposure to joy FEs increased adults’ subjective food liking. Therefore, exposure to others’ enjoyment of food (conveyed through FEs) influences the observer’s perceived liking and desirability of the food.

One food group that is often less preferred across the lifespan is vegetables, particularly those that are characterized by bitter tastes (e.g., broccoli; Wardle and Cooke, 2008; Dinnella et al., 2016; Hoffman et al., 2016). Exposure to eaters enjoying nutritious foods that are typically disliked, such as vegetables, could influence young adults’ vegetable consumption. Indeed, research in children has demonstrated that exposure to videos of adults eating raw broccoli with positive FEs increases children’s acceptance and intake of raw broccoli (Edwards et al., 2022). However, the effect of others’ FEs on young adults’ eating of vegetables remains to be examined.

It is also important to investigate whether modeling influences the desirability of foods that are not modeled. It is not yet clear whether modeling of a positive response to a vegetable influences the desirability of another similar vegetable, as well as the modeled food. Two vegetables which are similar in color and energy density, and which can both be served raw are broccoli and cucumber. Thus, based on the principles of classical conditioning, it is plausible that this perceived safety of one food will generalize to similar food (e.g., other vegetables). Establishing whether the effect of modeling generalises to a non-modeled vegetable will provide a greater theoretical understanding of the mechanisms underlying the modeling of eating. For example, if modeling occurs through observational learning of food safety or enjoyment (i.e., imitating the behavior of others; Bandura, 1977), generalization effects would not be expected. However, if it occurs by social facilitation of eating (i.e., adjusting eating behavior due to the presence of others; Herman, 2015), generalization to other foods would be predicted. Engagement in one health behavior can promote engagement in another health behavior (Dohle et al., 2015), thus the effect of others’ FEs could generalize to the eating of a non-modeled vegetable. However, this remains to be examined in adult populations. Examining the potential for generalization will elucidate the practicality of using modeling as a strategy to encourage young adults’ vegetable consumption more broadly.

This study examined the effect of models’ FEs toward raw broccoli on young adult women’s change in liking and change in desire to eat a modeled vegetable (raw broccoli) and a non-modeled vegetable (cucumber). Women were examined because gender differences may exist within the modeling of eating behavior, with larger modeling effects on women’s, than men’s, eating (Vartanian et al., 2015). Based on previous literature, it was hypothesized that there would be a greater increase in change in liking and desire to eat the modeled vegetable (raw broccoli) after exposure to videos of adult models consuming raw broccoli with positive FEs, and a greater decrease in change in liking and desire to eat the modeled vegetable after exposure to videos of adult models eating raw broccoli with negative FEs, compared to exposure to videos of adult models eating raw broccoli with neutral FEs. Furthermore, it was hypothesized that the effect of models’ positive FEs would generalize to women’s change in liking and desire to eat a non-modeled vegetable (cucumber).

## 2 Method

### 2.1 Participants

A G*Power calculation (Faul et al., 2007) to detect a main effect of condition with *d* = 0.45 (Barthomeuf et al., 2009), 80% power, and *α* = 0.05, recommended 190 participants. Between May and July 2020, 279 young adults (18–30 years old) were recruited in the United Kingdom via online advertisements through Aston University and social media. Participants were told that the study was investigating the relationship between emotions and food. Young adults with current or previous eating disorders, food allergies, or diabetes were excluded. Young adults who did not fit the age criteria (18–30 years old) or gender criteria (women participants only) were excluded. Aston University Research Ethics Committee (#1332) provided ethical approval. All participants provided informed consent.

### 2.2 Design

A between-subjects design was utilized. Using the randomize feature in Qualtrics, participants were randomly assigned to one of three conditions (positive, negative, or neutral), in which they were shown one of three videos (see section 2.3.3. for details).

### 2.3 Measures

#### 2.3.1 Outcome measures

Change in expected liking and change in desire to eat raw broccoli and cucumber were measured, separately. Raw broccoli and cucumber were selected as the modeled and non-modeled vegetables, respectively, due to their similarity in color and energy density, and since they can both be served in their raw form (cucumber = 15 kcal and broccoli = 35 kcal, per 100 g). Raw broccoli and cucumber have also been identified as tasting bitter to many individuals. Indeed, research has demonstrated that children with low sensitivity to bitter tastes consumed more bitter tasting vegetables (raw broccoli, cucumber, and olives), compared to children with high sensitivity to bitter tastes (Bell and Tepper, 2006). Six additional food items were included to disguise the study aims (apple, grapes, tortilla chips, crisps, chocolate, and cookies). Participants were shown the word of each food, in a randomized order, and rated their liking and desire to eat each food, pre- and post-manipulation. Expected food liking was measured on a 100 mm visual analog scale (VAS; Thomas et al., 2016), anchored to the left and right with ‘absent / no liking’ and ‘most liking you can ever imagine’. Participants rated how much they wanted to eat each individual food at that time, on a 10-point scale, for example 10 being ‘I have a great desire to eat raw broccoli’ and 0 being ‘I have no desire to eat raw broccoli’ (Barthomeuf et al., 2009, 2012). Change scores were computed by subtracting post- from pre-manipulation scores.

#### 2.3.2 Sample characteristics

Demographic and lifestyle information was gathered: gender, age, ethnicity, employment status, smoking status, and whether participants ate breakfast or lunch regularly (Thomas et al., 2016). Height and weight were self-reported, to calculate BMI. Information about food allergies, intolerances or medical conditions affecting eating behavior was assessed to exclude participants based on eligibility criteria. Participants reported their habitual intake (measured as the number of daily servings) and liking (measured using 100 mm VAS from ‘not and all’ and ‘very much’) of fruit, vegetables, junk food and sugar-sweetened beverages to establish whether conditions differed at baseline (Thomas et al., 2016). Baseline differences between conditions for hunger and mood state were examined using VAS anchored from ‘not at all’ to ‘very much’. VAS items included: alert, drowsy, light-headed, anxious, happy, nauseous, sad, withdrawn, faint, hungry, full, desire to eat and thirst (Thomas et al., 2016). Participants also completed questionnaires measuring individual characteristics: appetitive traits; general eating style; food neophobia; sensory processing; anxiety; empathy; and autistic traits (Supplementary material S1 for details). These traits differ between individuals and are associated with selective eating behaviors, thus were examined to check that participants did not differ in these measures between conditions.

#### 2.3.3 Experimental videos

Each experimental video (positive, negative, or neutral) comprised 8 clips of different unfamiliar adult models consuming raw broccoli with a positive FE (positive condition), negative FE (negative condition), or neutral FE (neutral condition), presented in a randomized order. Videos had no sound and were intentionally short to avoid boredom effects (video length: positive = 84 s; negative = 77 s; neutral = 60 s). Each experimental video (positive, negative, or neutral) featured the same 8 models: 4 men and 4 women (aged 24–30 years) of different ethnicities (White British = 6; Asian British = 2). A pilot study with 20 adults, and FaceReader 7.0 software (Noldus, 2016) confirmed that each video conveyed the intended valence. Positive, negative, and neutral FEs were determined using overall valence scores, calculated using FaceReader 7.0 software (Noldus, 2016): the intensity of positive emotion (happy) minus the intensity of negative emotions (sad, angry, scared and disgust). Valence scores are categorized into positive (scores above 0.33), negative (scores below −0.33) or neutral (scores between −0.33 and 0.33). Results showed that each experimental video conveyed the intended valence of positive (*M* = 0.61, SD = 0.08; positive condition), negative (*M* = −0.57, SD = 0.22; negative condition), or neutral FEs (*M* = −0.12, SD = 0.07; control condition). For an example video clip of an adult model eating raw broccoli with a positive, negative, and neutral FE, please see https://doi.org/10.17036/researchdata.aston.ac.uk.00000552.

#### 2.3.4 Experimental task

Participants were told they would watch a video of adults eating raw broccoli and were instructed to watch the full video closely as they would be asked questions about it later. After watching the positive, negative, or neutral video, participants were asked to rate the valence of models’ FEs (positive, negative, or neutral), the authenticity of models’ FEs (genuine, pretend, not sure), how they thought the model actually felt (positive, negative or neutral), and the intensity of this feeling (100 mm VAS from ‘negative’ to ‘positive’).

### 2.4 Procedure

Through the online platform Qualtrics, participants provided consent, and demographic and lifestyle information. Participants then rated their liking and desire to eat broccoli, cucumber, and the 6 additional food items. Mood and hunger ratings were also completed. Next, participants completed the experimental task, followed by non-food related questionnaires examining autistic and empathetic traits. Participants completed post-manipulation ratings of their liking and desire to eat broccoli, cucumber and the 6 additional food items, and then questionnaires assessing habitual intake and liking, sensory processing, food neophobia, anxiety, appetitive traits and eating style. Finally, participants reported their height and weight. Participants were asked to guess the study aims and were debriefed and thanked for participating. The study took approximately 20 min to complete. Participants could enter a prize draw for a £50 shopping voucher for participating.

### 2.5 Statistical analysis

Data were analyzed using SPSS Version 26. Chi-square tests examined differences between conditions on ethnicity. One-way ANOVA examined differences between conditions for: demographics, habitual food intake and liking, mood and hunger scores, and questionnaires measuring individual characteristics. Measures that differed significantly between conditions were included as covariates in main analyses. Baseline liking and desire to eat scores for broccoli and cucumber were included in ANCOVA when change in liking and change in desire to eat were the outcome measures (Clifton and Clifton, 2019). One-way ANCOVA examined main effects of condition on dependent variables. Bonferroni corrected *t*-tests followed up significant main effects of condition.

## 3 Results

### 3.1 Demographics and baseline measures

In total, 279 young adults completed the study. Participants were excluded for: having a food allergy (*n* = 9); not meeting age inclusion criteria (*n* = 1); not meeting gender inclusion criteria (*n* = 40); and not identifying the correct FE for their condition, as this indicated they might not have watched the video manipulation, given the online nature of the study (*n* = 24). The final sample included 205 young adult women. Participants mean age was 22.3 years (SD = 2.64; range = 18–30) and mean BMI was in the healthy range (*M* = 23.50, SD = 4.69). Participant’s ethnicity was 42.4% White British, 11.7% Indian, 11.7% Pakistani, 3.9% Bangladeshi, 3.4% Chinese, 3.4% White and Asian, 2.9% Black African, 2.4% Black Caribbean, 1.5% White and Black Caribbean, 1.0% White and Black African, 0.5% White Irish, 14.1% ‘other’ and 1.0% ‘prefer not to say’. Participants were mainly students (79.5% full-time; 5.4% part-time) and unemployed (48.8%) or employed part-time (34.1%). Most participants ate breakfast (65.4%) and lunch regularly (85.4%) and did not smoke (96.6%).

Ethnicity was not significantly different between conditions (*X^2^*(205) = 26.32, *p* = 0.34). Demographic measures and baseline food intake and liking scores did not differ significantly between conditions (Table 1). Baseline mood and appetite scores were not significantly different between conditions (all *p*’s > 0.05, Supplementary Table S1). Finally, questionnaires measuring individual characteristics showed no significant differences between conditions (all *p*’s > 0.05; Supplementary Table S2). See Supplementary Table S3 for mean change in liking and change in desire to eat scores for additional food items used to disguise the study aims.

**Table 1:** Mean (SD) demographics and baseline food intake and liking scores split by condition (one-way ANOVA).

	Positive (*n* = 65)	Negative (*n* = 73)	Neutral (*n* = 67)	Range	*F*	*p*
Age	22.54 (2.50)	22.59 (2.83)	21.84 (2.52)	18–30	1.74	0.18
BMI	24.58 (4.60)	22.98 (5.20)	23.00 (5.20)	15.6–46.3	2.60	0.08
Habitual fruit intake	2.32 (2.32)	1.98 (1.23)	1.93 (1.09)	0–10	1.77	0.17
Habitual vegetable intake	2.52 (1.81)	2.73 (1.93)	2.25 (1.20)	0–12	1.46	0.23
Habitual junk food intake	1.95 (1.37)	1.74 (0.99)	1.73 (1.18)	0–6	0.74	0.48
Habitual SSB intake	1.02 (1.19)	1.14 (1.46)	0.70 (1.05)	0–9	2.24	0.11
Habitual fruit liking	83.45 (19.04)	78.03 (22.17)	79.90 (18.46)	10–100	1.28	0.28
Habitual vegetable liking	71.69 (23.60)	74.32 (24.00)	70.15 (23.54)	0–100	0.56	0.57
Habitual junk food liking	79.40 (20.45)	76.52 (19.57)	77.79 (25.35)	0–100	0.30	0.74
Habitual SSB liking	60.22 (32.97)	61.38 (28.50)	53.87 (35.06)	0–100	1.08	0.34
Broccoli Liking	30.35 (28.47)	31.27 (25.80)	30.27 (25.80)	0–100	0.03	0.97
Cucumber liking	65.54 (27.67)	66.07 (24.98)	66.13 (27.94)	0–100	0.01	0.99
Desire to eat broccoli	2.18 (2.28)	2.16 (2.53)	2.27 (2.47)	0–9	0.04	0.97
Desire to eat cucumber	5.77 (2.96)	5.74 (2.92)	5.99 (3.09)	0–10	0.14	0.87

Age = years; Habitual intake = number of daily servings; Habitual liking = ratings on a 100 mm VAS from ‘not and all’ and ‘very much’; Liking = ratings on a 100 mm VAS from ‘no liking’ to ‘most liking you can ever imagine’; Desire to eat = ratings on a 10-point scale from ‘I have a great desire to eat’ to ‘I have no desire to eat’ SSB = Sugar-sweetened beverages.

### 3.2 Manipulation check

Most participants thought that the models’ FEs they observed were pretend (positive = 87.7%; negative = 79.5%; neutral = 68.7%), and few participants (16.9%) in the positive condition thought models felt positive about eating raw broccoli. Most participants in the negative condition (56.2%) thought models felt negative about eating raw broccoli, and most participants in the neutral condition (52.2%) thought models felt neutral about eating raw broccoli. Positive and negative FEs were rated as higher in intensity (*M* = 69.49 and *M* = 80.19, respectively), and neutral FEs were rated as lower in intensity (*M* = 36.51). Excluding participants who guessed the study aims (*n* = 12) did not change the effect of condition on dependent variables. Therefore, all cases were included in analyses.

### 3.3 Change in liking

**Figure 1:**
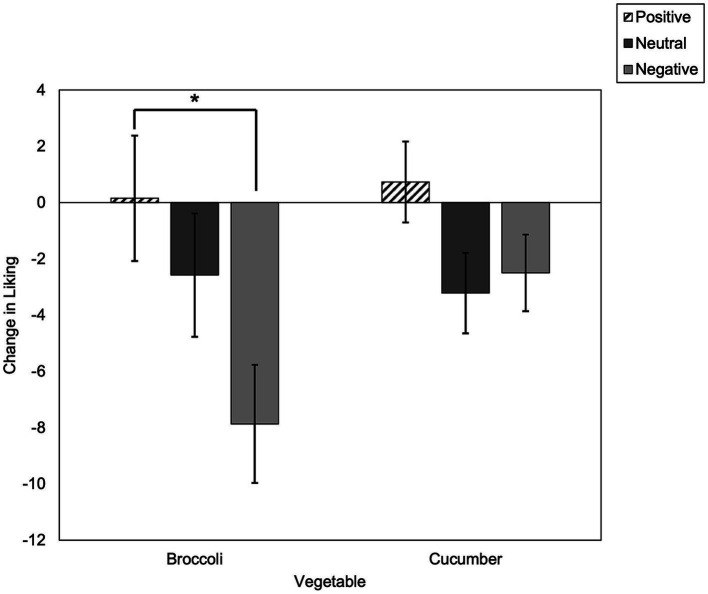
Estimated marginal means of change in vegetable liking scores split by condition (standard error). *p < 0.05.

ANCOVA, controlling for baseline broccoli liking, revealed there was a significant main effect of condition on the change in broccoli liking [*F* (2, 201) = 3.60, *p* = 0.03, *η*_p_^2^ = 0.04; Figure 1]. Bonferroni corrected *t*-tests revealed that the change in broccoli liking was significantly more negative in the negative condition, compared to the positive condition (*p* = 0.03). Change in broccoli liking did not differ significantly between neutral vs. positive, or neutral vs. negative conditions (*p*’s > 0.05). ANCOVA, controlling for baseline cucumber liking, revealed there was no significant main effect of condition on the change in cucumber liking [*F* (2, 201) = 2.16, *p* = 0.12, *η*_p_^2^ = 0.02].

### 3.4 Change in desire to eat

ANCOVA, controlling for baseline desire to eat scores, revealed there was no significant main effect of condition on the change in desire to eat broccoli [*F* (2, 201) = 1.32, *p* = 0.27, *η*_p_^2^ = 0.01] and cucumber [*F* (2, 201) = 1.16, *p* = 0.32, *η*_p_^2^ = 0.01; Table 2].

**Table 2:** Estimated marginal means (standard error) of change in vegetable desire to eat scores split by condition.

	Positive (*n* = 65)	Negative (*n* = 73)	Neutral (*n* = 67)
Desire to eat broccoli	0.07 (0.20)	−0.34 (0.19)	0.03 (0.20)
Desire to eat cucumber	0.03 (0.16)	0.05 (0.15)	−0.26 (0.16)

## 4 Discussion

This study examined the effect of models’ FEs while consuming raw broccoli, on women’s change in liking and desire to eat a modeled vegetable (raw broccoli) and a non-modeled vegetable (cucumber). Partially supporting our hypotheses, exposure to models eating broccoli whilst conveying negative FEs resulted in a greater reduction in liking ratings of the modeled vegetable, compared to the positive condition. However, this effect did not generalize to the non-modeled vegetable. Furthermore, positive FEs had no significant effect on liking, compared to the neutral condition, and participants’ desire to eat any of the foods was not related to the manipulation. Hence, the findings suggest a selective effect, whereby exposure to negative FEs reduces the liking of the modeled vegetable.

One explanation of why the change in broccoli liking was more negative after exposure to models eating broccoli with negative FEs, is that avoiding foods associated with disgust is an adaptive response, to prevent ingestion of harmful substances (Curtis, 2011). This has concerning implications for the liking of nutritious foods. For example, observing someone showing dislike whilst eating a vegetable could decrease the observers’ liking of this vegetable. However, this effect does not appear to generalize to the liking of other vegetables. As omnivores, our diet would be highly restricted if we excluded all similar foods that we saw someone once disliking. Since broccoli and cucumber look different, the absence of a generalized effect could be explained by individuals paying particular attention to exactly what the model is eating, and their behavior inhibits ingestion only of that food. Another explanation for the absence of a generalized effect could be participants’ familiarity with the vegetables. For example, cucumber is likely to be more common than broccoli, when served raw (broccoli is often served cooked). Furthermore, all participants were exposed to broccoli as the modeled vegetable and cucumber as the non-modeled vegetable. Therefore, the lack of a generalized effect could be due to differences in palatability between the vegetables. Indeed, at baseline, liking and desire to eat cucumber was higher than participant’s liking and desire to eat broccoli. Thus, future research examining the generalized effect of exposure to models’ FEs whilst eating should include vegetables of similar familiarity and palatability. Research in real-life eating occasions is needed to examine the effect of observing another eater show dislike whilst eating nutritious food on the observers’ actual intake of these foods.

Contrary to hypotheses, exposure to models eating broccoli with positive FEs, did not result in a greater increase in change in liking or desire to eat ratings for any foods. One explanation for this could be that positive FEs do not demonstrate food enjoyment convincingly. Indeed, few participants in the positive condition thought that the models really felt positive about eating broccoli, potentially because smiling whilst eating is not a typical reaction to liked tastes. Instead, naturalistically, liked tastes elicit low intensity, relaxed and soothed facial responses (Wendin et al., 2011). Therefore, displaying exaggerated smiles whilst eating a vegetable might not accurately convey food enjoyment to other adults. In contrast, disliked tastes do elicit intense, disgust-like facial responses (Horio, 2003; Wendin et al., 2011; Danner et al., 2014) and in the negative condition over half of participants believed the models did not enjoy eating the broccoli. Another explanation could be that the risk associated with ingesting a disgusting food is greater than the possibility of enjoying a food and thus we pay more attention to, or are more likely to, adjust our behavior in response to, negative FEs. Avoiding disgusting foods is protective (Curtis, 2011), thus participants might have been more inclined to adjust their eating behavior after seeing a negative FE, rather than a positive FE, to protect themselves from harm. Therefore, it is possible that reducing food liking could be easier to achieve than increasing food liking.

A further interesting finding was that subjective food liking was influenced by models’ FEs, but desire to eat was not, which contradicts our predictions and previous research (Barthomeuf et al., 2009, 2012). Although there was possibility to reduce eating desire in this study, the low baseline desire to eat might explain the absence of a significant effect of negative FEs on participant’s desire to eat broccoli. Consistent with previous research, exposure to negative FEs does not reduce the eating desire of food that participants already have a low desire to eat (Rousset et al., 2008).

The current findings demonstrate that positive FEs do not appear effective for influencing young adult women’s subjective vegetable liking and eating desire. However, the models’ positive FEs were commonly perceived to be pretend, which could have influenced their effect on eating behavior. Importantly, these pretend positive FEs did not have negative effects on participants’ eating behavior, suggesting that they were merely not helpful, rather than harmful. Furthermore, almost half of participants in the positive condition did not correctly identify how models felt about eating the broccoli. Therefore, the models’ positive FEs might not have accurately conveyed food enjoyment, which might have reduced the effectiveness of the manipulation. Moreover, since positive FEs were often perceived to be pretend, participants might not have used the models’ FEs as useful information to indicate the palatability of the vegetables. Future research should expose participants to real-life food enjoyment, to establish whether naturalistic facial reactions whilst eating influence young adults’ eating behavior. Additionally, the measurement of subjective wanting and liking is limited. For example, subjective ratings of desire to eat might not reflect participants’ actual motivational desire to consume the food. Participants rated their eating desire based on seeing the word (e.g., ‘broccoli’), rather than seeing an image of broccoli, or the vegetable itself, which would have been more ecologically valid. However, the online data collection meant that food intake could not be directly and objectively measured. Research that objectively measured facial reactions (using electromyography) found that exposure to videos of avatars looking at palatable food with positive FEs increased adults’ positive facial reactions (zygomatic activity) but did not change their subjective ratings of liking and wanting (Soussignan et al., 2019). Thus, future research should objectively measure participants’ facial reactions (e.g., using electromyography or FaceReader software). While expected food liking has been found to be an important and malleable predictor of young adults’ food choice (Robinson et al., 2012, 2013; Robinson and Higgs, 2012), research measuring food intake is needed to determine whether reductions in food liking translate to actual food consumption.

This study was sufficiently powered to detect a small to medium effect size, using an efficient experimental design. The current findings are only relevant to women. Though research has suggested larger modeling effects for women (Vartanian et al., 2015), it is unclear whether gender would interact with the effect of models’ FEs on eating behavior, thus, future research is needed with samples comprising sufficient numbers of men. It is also possible that the gender of the models could have moderated the effect of FEs on eating behavior. Whilst this cannot be examined in the current study, all participants were exposed to a range of models so that participants could identify with some of them. Whilst individual characteristics were assessed in this study to ensure matching of participants between experimental conditions on variables known to influence eating behavior and/ or emotion perception, further work with larger samples could examine whether these characteristics, such as empathy, might moderate the effect of others’ FEs on eating behavior. Moreover, the findings could have been influenced by demand characteristics. Whilst a cover story was given, participants might have adjusted their eating behavior in response to their beliefs about the true aims of the study. One noteworthy strength was the novel use of video stimuli, which allowed participants to observe the dynamic nature of others’ FEs whilst eating. This method is more ecologically valid than the previously used static images of models (Barthomeuf et al., 2009, 2012).

In conclusion, this study demonstrated that exposing young adult women to videos of others expressing negative FEs whilst eating raw broccoli, decreased their liking of the modeled vegetable, but not the non-modeled vegetable, or their desire to consume either vegetable. This highlights the power of negative FEs toward food in reducing food liking. Further work is needed to establish whether observing specific FEs shown by models eating nutritious foods influences actual vegetable consumption, and whether acute changes in liking have a long-term effect on eating behavior. Research examining whether gender interacts with the effect of modeling on eating behavior is required. Investigating these questions will help to further understand the effect of exposure to others’ FEs on young adults’ vegetable consumption.

## Data availability statement

The raw data supporting the conclusions of this article data will be made available upon request by the authors, without undue reservation.

## Ethics statement

The studies involving humans were approved by the Aston University Research Ethics Committee (#1332). The studies were conducted in accordance with the local legislation and institutional requirements. The participants provided their written informed consent to participate in this study.

## Author contributions

KE conducted the research, analyzed data, and drafted the primary manuscript. JT, SH, and JB contributed to the writing of the manuscript and its editing. All authors contributed to the design of the research, manuscript revision, read, and approved the submitted version.
