# Supplementary material for: Exposure to models’ negative facial expressions whilst eating a vegetable decreases women’s liking of the modelled vegetable, but not their desire to eat
Source: Front Psychol. 2024 Jan 11;14:1252369. doi: 10.3389/fpsyg.2023.1252369 (PMC10808660; doi:10.3389/fpsyg.2023.1252369)
Supplement: Supplementary file 1 [file Data_Sheet_1.docx]

**Questionnaires measuring individual characteristics**

***Adult’s Eating Behaviour Questionnaire (AEBQ; (Hunot et al., 2016)***

The AEBQ measured participants’ appetitive traits on 8 subscales: enjoyment of food (3 items, e.g., ‘I love food’); emotional over-eating (5 items, e.g., ‘I eat more when I’m annoyed’); emotional under-eating (5 items, e.g., ‘I eat less when I’m worried’); food fussiness (5 items, e.g., ‘I refuse new foods at first’); food responsiveness (4 items, e.g., ‘I am always thinking about food’); slowness in eating (4 items, e.g., ‘I eat slowly’); hunger (5 items, e.g., ‘I often feel hungry’); and satiety responsiveness (4 items, e.g., ‘I get full up easily’). Responses are on a 4-point liking scale from 1 (strongly disagree) to 5 (strongly agree). The AEBQ is a reliable and valid measure (Hunot et al., 2016; Hunot-Alexander et al., 2019) and subscales showed good internal consistency in this study (α = 0.67 – 0.89).

***Adolescent / Adult Sensory Profile (AASP; Brown & Dunn, 2002)***

Three subscales of the AASP measured participant’s sensory processing: taste/smell processing (8-items, e.g., ‘I add spice to my food’); visual processing (10-items, e.g., ‘I don’t notice when people come into the room’); and touch processing (13-items, e.g., ‘I like how it feels to get my hair cut’). Responses are on a 5-item Likert scale from ‘almost never’ to ‘almost always’. Sensory sensitivity is associated with picky eating (Zickgraf & Elkins, 2018) and greater intake of unhealthier palatable foods (Naish & Harris, 2012). Thus, differences between conditions in sensory processing domains were examined (low registration, sensory sensitivity, sensation seeking, sensory avoiding). The AASP has satisfactory internal consistency and discriminant validity (Brown & Dunn, 2002) and showed satisfactory internal consistency in this sample (α = 0.44 – 0.57).

***Autistic-Spectrum Quotient (AQ-10; (Allison et al., 2012)***

The 10-item version of the Autism-Spectrum Quotient measured participants’ autistic traits (e.g., ‘I find it difficult to work out people’s intentions’). Responses are scored as 1 and 0, indicating autistic traits or not, respectively. Differences in autistic traits between conditions were examined since Autism Spectrum Disorder is characterised by a deficit in facial processing (Webb et al., 2017), and is associated with food selectivity (Mari-Bauset et al., 2014; Spek et al., 2020). The AQ-10 has good internal reliability, excellent predictive validity and correlates with the 50-item Austism-Spectrum Quotient (Allison et al., 2012). However, internal consistency in this sample was below acceptable (α = 0.43).

***Beck’s Anxiety Inventory (BAI; (Beck et al., 1988)***

Somatic symptoms of anxiety were measured using BAI. Individuals report how much a symptom bothers them using a 4-point Likert scale (e.g., ‘numbness’ on a scale from ‘not at all’ to ‘severely’). Anxiety has been associated with picky eating behaviour (Fox et al., 2018; Wildes et al., 2012; Zickgraf & Elkins, 2018), thus, differences in anxiety between conditions were assessed. The BAI has good internal consistency, test-retest reliability, and robust convergent reliability (Bardhoshi et al., 2016). Internal consistency in this study was excellent (α = 0.93).

***Food Neophobia Scale (FNS; (Pliner & Hobden, 1992)***

The 10-item FNS measured participants’ willingness to try novel foods on a 7-point Likert scale ranging from ‘disagree strongly’ to ‘agree strongly’ (e.g., ‘I don’t trust new foods’). Food neophobia is associated with picky eating (Elkins & Zickgraf, 2018; Jaeger et al., 2017) and lower fruit and vegetable consumption (Costa et al., 2020; Knaapila et al., 2015), thus differences in FNS scores between conditions were examined. The FNS has good internal reliability and test-retest reliability (Pliner & Hobden, 1992). It also has good predictive validity, as scores predicted behaviour to novel foods in laboratory tasks, and good convergent and discriminant validity (Pliner & Hobden, 1992). Internal consistency in this study was good (α = 0.80).

***Three Factor Eating Questionnaire (TFEQ-R21; (Cappelleri et al., 2009)***

Participants’ eating style was measured using the TFEQ-R21: cognitive restraint (6 items, e.g., ‘I deliberately take small helpings to control my weight’); uncontrolled eating (9 items, e.g., ‘sometimes when I start eating, I just can’t seem to stop’); and emotional eating (6 items, e.g., ‘when I feel lonely, I console myself by eating’). Responses are on a 4-point Likert scale (i.e., ‘definitely true’ to ‘definitely false’). Eating style was assessed to characterise the sample and to examine associations with outcome measures. The TFEQR-21 is a commonly used measure and has been validated in obese and non-obese populations, showing good psychometric properties (Cappelleri et al., 2009). Subscales had good internal consistency in this study (α = 0.82 – 0.92).

***Toronto Empathy Scale (TEQ; (Spreng et al., 2009)***

Participants’ empathy was measured using the 16-item TEQ (e.g., ‘I enjoy making other people feel better’). Responses are on a 5-point Likert scale ranging from ‘never’ to ‘always’. High empathy is associated with greater modelling of eating behaviour, thus differences between conditions were assessed (Robinson et al., 2011). The TEQ has good internal reliability, test-retest reliability, and convergent validity (Spreng et al., 2009). Internal consistency in this study was good (α = 0.81).

**Supplementary Tables**

**Supplemental Table 1:** Mean (SD) baseline mood and hunger scores for participants in each condition (one-way ANOVA)

|  | Positive | Negative | Neutral | *F* | *p* |
| --- | --- | --- | --- | --- | --- |
| Alertness | 59.22 (21.37) | 64.15 (21.99) | 65.01 (20.60) | 1.42 | .24 |
| Drowsiness | 47.71 (28.90) | 40.48 (24.80) | 37.46 (28.63) | 2.43 | .09 |
| Light-headed | 25.20 (25.65) | 22.10 (20.00) | 24.04 (26.87) | 0.29 | .75 |
| Anxious | 38.38 (30.93) | 37.12 (29.31) | 40.06 (31.33) | 0.16 | .85 |
| Happiness | 63.23 (20.16) | 64.58 (20.70) | 67.19 (17.39) | 0.71 | .49 |
| Nausea | 17.23 (21.39) | 15.49 (22.90) | 15.48 (23.49) | 0.13 | .88 |
| Sadness | 34.00 (28.33) | 33.32 (26.72) | 31.93 (27.48) | 0.10 | .91 |
| Withdrawn | 32.00 (29.14) | 32.32 (28.28) | 27.18 (26.34) | 0.72 | .49 |
| Faint | 12.63 (15.89) | 14.01 (19.02) | 13.76 (21.61) | 0.10 | .90 |
| Energised | 44.15 (24.69) | 47.36 (22.44) | 51.30 (24.89) | 1.47 | .23 |
| Stressed | 49.69 (26.21) | 42.18 (31.38) | 49.16 (28.94) | 1.47 | .23 |
| Hunger | 37.45 (32.04) | 36.81 (29.87) | 28.91 (27.69) | 1.70 | .19 |
| Fullness | 59.02 (28.42) | 53.07 (27.74) | 59.78 (29.70) | 1.16 | .31 |
| Desire to Eat | 46.11 (31.95) | 27.08 (29.92) | 42.91 (30.16) | 0.35 | .71 |
| Thirst | 61.41 (24.66) | 56.37 (24.88) | 61.36 (24.73) | 0.95 | .39 |

**Supplemental Table 2:** Mean (SD) scores on questionnaires measuring individual characteristics for participants in each condition (one-way ANOVA)

|  | Positive | Negative | Neutral | *F* | *p* |
| --- | --- | --- | --- | --- | --- |
| **AASP** |  |  |  |  |  |
| Low Registration | 14.25 (3.93) | 14.51 (3.56) | 14.64 (3.56) | 0.20 | .82 |
| Sensation Seeking | 26.05 (4.11) | 25.21 (4.68) | 25.82 (3.64) | 0.76 | .47 |
| Sensory Sensitivity | 20.75 (4.77) | 20.49 (4.24) | 20.48 (4.89) | 0.08 | .93 |
| Sensory Avoiding | 21.31 (4.50) | 21.55 (4.13) | 21.97 (4.93) | 0.37 | .70 |
| **AEBQ** |  |  |  |  |  |
| Enjoyment of food | 4.37 (0.67) | 4.20 (0.73) | 4.41 (0.71) | 1.82 | .16 |
| Emotional overeating | 2.88 (1.02) | 2.53 (1.04) | 2.50 (0.95) | 2.88 | .06 |
| Emotional undereating | 3.08 (1.02) | 3.32 (0.95) | 3.36 (0.93) | 1.55 | .22 |
| Food fussiness | 2.30 (0.93) | 2.30 (0.93) | 2.41 (0.95) | 0.32 | .73 |
| Food responsiveness | 3.41 (0.79) | 3.30 (0.76) | 3.38 (0.71) | 0.39 | .68 |
| Hunger | 3.03 (0.76) | 3.08 (0.76) | 3.09 (0.71) | 0.12 | .89 |
| Slowness in eating | 2.89 (0.89) | 2.76 (1.06) | 2.69 (1.03) | 0.72 | .49 |
| Satiety responsiveness | 2.57 (0.75) | 2.73 (0.84) | 2.82 (0.80) | 1.73 | .18 |
| **AQ-10** | 3.12 (1.71) | 3.16 (1.92) | 3.03 (1.69) | 0.10 | .90 |
| **BAI** | 18.08 (10.43) | 18.36 (11.72) | 18.51 (12.65) | 0.02 | .98 |
| **FNS** | 33.29 (10.24) | 35.00 (9.81) | 35.03 (9.83) | 0.66 | .52 |
| **TEQ** | 49.22 (6.30) | 49.68 (6.82) | 50.22 (5.78) | 0.42 | .66 |
| **TFEQR-21** |  |  |  |  |  |
| Uncontrolled eating | 21.32 (5.46) | 20.33 (5.34) | 20.39 (4.71) | 0.77 | .46 |
| Cognitive restraint | 13.85 (3.62) | 14.42 (4.35) | 14.62 (4.46) | 0.61 | .55 |
| Emotional eating | 14.77 (4.93) | 12.81 (5.34) | 13.19 (4.73) | 2.88 | .06 |

**Note.** Adolescent/Adult Sensory Profile (AASP); Adult Eating Behaviour Questionnaire (AEBQ); Autism-Spectrum Quotient (AQ-10); Beck’s Anxiety Inventory (BAI); Food Neophobia Scale (FNS); Toronto Empathy Questionnaire (TEQ); Three Factor Eating Questionnaire (TFEQ-R21).

**Supplemental Table 3:** Mean (SD) change in liking and change in desire to eat scores for additional food items

|  |  | Mean (SD) |
| --- | --- | --- |
| Change in liking | Grapes | -1.19 (11.91) |
|  | Apple | .50 (12.41) |
|  | Crisps | -.98 (12.02) |
|  | Tortilla chips | -3.33 (14.06) |
|  | Chocolate | -.21 (10.51) |
|  | Cookies | -1.34 (13.49) |
| Desire to eat | Grapes | -.01 (1.34) |
|  | Apple | .05 (1.45) |
|  | Crisps | -.08 (1.37) |
|  | Tortilla chips | .01 (1.51) |
|  | Chocolate | -.08 (1.63) |
|  | Cookies | -.12 (1.53) |

**Supplementary References**

Allison, C., Auyeung, B., & Baron-Cohen, S. (2012). Toward Brief "Red Flags" for Autism Screening: The Short Autism Spectrum Quotient and the Short Quantitative Checklist for Autism in Toddlers in 1,000 Cases and 3,000 Controls (vol 51, pg 202, 2011 ). *Journal of the American Academy of Child and Adolescent Psychiatry*, *51*(3), 338-338. <https://doi.org/10.1016/j.jaac.2012.01.017>

Bardhoshi, G., Duncan, K., & Erford, B. T. (2016). Psychometric Meta-Analysis of the English Version of the Beck Anxiety Inventory. *Journal of Counseling and Development*, *94*(3), 356-373. <https://doi.org/10.1002/jcad.12090>

Beck, A. T., Brown, G., Epstein, N., & Steer, R. A. (1988). An inventory for measuring clinical anxiety - psychometric properties. *Journal of Consulting and Clinical Psychology*, *56*(6), 893-897. <https://doi.org/10.1037/0022-006x.56.6.893>

Brown, C., & Dunn, W. (2002). Adolescent/adult sensory profile. San Antonio, TX: Psychological Corporation.

Cappelleri, J. C., Bushmakin, A. G., Gerber, R. A., Leidy, N. K., Sexton, C. C., Lowe, M. R., & Karlsson, J. (2009). Psychometric analysis of the Three-Factor Eating Questionnaire-R21: results from a large diverse sample of obese and non-obese participants. *International Journal of Obesity*, *33*(6), 611-620. <https://doi.org/10.1038/ijo.2009.74>

Costa, A., Silva, C., & Oliveira, A. (2020). Food neophobia and its association with food preferences and dietary intake of adults. *Nutrition & Dietetics*, *77*(5), 542-549. <https://doi.org/10.1111/1747-0080.12587>

Elkins, A., & Zickgraf, H. F. (2018). Picky eating and food neophobia: Resemblance and agreement in parent/young adult dyads. *Appetite*, *126*, 36-42. <https://doi.org/10.1016/j.appet.2018.02.021>

Fox, G., Coulthard, H., Williamson, L., & Wallis, D. (2018). "It's always on the safe list": Investigating experiential accounts of picky eating adults. *Appetite*, *130*, 1-10. <https://doi.org/10.1016/j.appet.2018.07.023>

Hunot, C., Fildes, A., Croker, H., Llewellyn, C. H., Wardle, J., & Beeken, R. J. (2016). Appetitive traits and relationships with BMI in adults: Development of the Adult Eating Behaviour Questionnaire. *Appetite*, *105*, 356-363. <https://doi.org/10.1016/j.appet.2016.05.024>

Hunot-Alexander, C., Beeken, R. J., Goodman, W., Fildes, A., Croker, H., Llewellyn, C., & Steinsbekk, S. (2019). Confirmation of the Factor Structure and Reliability of the 'Adult Eating Behavior Questionnaire' in an Adolescent Sample. *Frontiers in Psychology*, *10*, Article 1991. <https://doi.org/10.3389/fpsyg.2019.01991>

Jaeger, S. R., Rasmussen, M. A., & Prescott, J. (2017). Relationships between food neophobia and food intake and preferences: Findings from a sample of New Zealand adults. *Appetite*, *116*, 410-422. <https://doi.org/10.1016/j.appet.2017.05.030>

Knaapila, A. J., Sandell, M. A., Vaarno, J., Hoppu, U., Puolimatka, T., Kaljonen, A., & Lagstrom, H. (2015). Food neophobia associates with lower dietary quality and higher BMI in Finnish adults. *Public Health Nutrition*, *18*(12), 2161-2171. <https://doi.org/10.1017/s1368980014003024>

Mari-Bauset, S., Zazpe, I., Mari-Sanchis, A., Llopis-Gonzalez, A., & Morales-Suarez-Varela, M. (2014). Food Selectivity in Autism Spectrum Disorders: A Systematic Review. *Journal of Child Neurology*, *29*(11), 1554-1561. <https://doi.org/10.1177/0883073813498821>

Naish, K. R., & Harris, G. (2012). Food Intake Is Influenced by Sensory Sensitivity. *Plos One*, *7*(8), Article e43622. <https://doi.org/10.1371/journal.pone.0043622>

Pliner, P., & Hobden, K. (1992). DEVELOPMENT OF A SCALE TO MEASURE THE TRAIT OF FOOD NEOPHOBIA IN HUMANS. *Appetite*, *19*(2), 105-120. <https://doi.org/10.1016/0195-6663(92)90014-w>

Robinson, E., Tobias, T., Shaw, L., Freeman, E., & Higgs, S. (2011). Social matching of food intake and the need for social acceptance. *Appetite*, *56*(3), 747-752. <https://doi.org/10.1016/j.appet.2011.03.001>

Spek, A. A., van Rijnsoever, W., van Laarhoven, L., & Kiep, M. (2020). Eating Problems in Men and Women with an Autism Spectrum Disorder. *Journal of Autism and Developmental Disorders*, *50*(5), 1748-1755. <https://doi.org/10.1007/s10803-019-03931-3>

Spreng, R. N., McKinnon, M. C., Mar, R. A., & Levine, B. (2009). The Toronto Empathy Questionnaire: Scale Development and Initial Validation of a Factor-Analytic Solution to Multiple Empathy Measures. *Journal of Personality Assessment*, *91*(1), 62-71, Article Pii 906598365. <https://doi.org/10.1080/00223890802484381>

Webb, S. J., Neuhaus, E., & Faja, S. (2017). Face perception and learning in autism spectrum disorders. *Quarterly Journal of Experimental Psychology*, *70*(5), 970-986. <https://doi.org/10.1080/17470218.2016.1151059>

Wildes, J. E., Zucker, N. L., & Marcus, M. D. (2012). Picky eating in adults: Results of a web-based survey. *International Journal of Eating Disorders*, *45*(4), 575-582. <https://doi.org/10.1002/eat.20975>

Zickgraf, H. F., & Elkins, A. (2018). Sensory sensitivity mediates the relationship between anxiety and picky eating in children/adolescents ages 8-17, and in college undergraduates: A replication and age-upward extension. *Appetite*, *128*, 333-339. <https://doi.org/10.1016/j.appet.2018.06.023>
